# Ultrasound-Guided Inactivation of Trigger Points Combined with Muscle Fascia Stripping by Liquid Knife in Treatment of Postherpetic Neuralgia Complicated with Abdominal Myofascial Pain Syndrome: A Prospective and Controlled Clinical Study

**DOI:** 10.1155/2020/4298509

**Published:** 2020-05-17

**Authors:** Xiang-Hong Lu, Xiao-Lan Chang, Si-Lan Liu, Jing-Ya Xu, Xiao-Jun Gou

**Affiliations:** ^1^Anesthesia Surgery Department, The First Affiliated Hospital of Suzhou University, Suzhou 215006, China; ^2^Central Laboratory, Baoshan District Hospital of Integrated Traditional Chinese and Western Medicine of Shanghai, Shanghai 201999, China

## Abstract

**Objective:**

To evaluate ultrasound-guided inactivation of myofascial trigger points (MTrPs) combined with abdominal muscle fascia stripping by liquid knife in the treatment of postherpetic neuralgia (PHN) complicated with abdominal myofascial pain syndrome (AMPS).

**Methods:**

From January 2015 to July 2018, non-head-and-neck PHN patients in the Pain Department, The First Affiliated Hospital of Soochow University, were treated with routine oral drugs and weekly paraspinal nerve block for two weeks. Patients with 2 < VAS (visual analogue scale) score < 6 were subjects of the study. They were assigned into control group 1 (C1, *n* = 33) including those with PHN and without myofascial pain syndrome (MPS) and control group 2 (C2, *n* = 33) including those with PHN complicated with MPS and observation group 1 (PL, *n* = 33) including those with PHN complicated with limb myofascial pain syndrome (LMPS) and observation group 2 (PA, *n* = 33) including those with PHN complicated with AMPS. All groups received zero-grade treatment: routine oral drugs and weekly paraspinal nerve block. PL and PA groups were also treated step by step once a week: primary ultrasound-guided inactivation of MTrPs with dry needling, secondary ultrasound-guided inactivation of MTrPs with dry and wet needling, and tertiary ultrasound-guided dry and wet needling combined with muscle fascia stripping by liquid knife. At one week after primary treatment, patients with a VAS score > 2 proceeded to secondary treatment. If the VAS score was <2, the treatment was maintained, and so on, until the end of the four treatment cycles. Pain assessment was performed by specialized nurses at one week after each treatment, including VAS score, McGill pain questionnaire (MPQ) score, pressure pain sensory threshold (PPST), and pressure pain tolerance threshold (PPTT). VAS score was used as the main index and VAS <2 indicated effective treatment. At 3 months after treatment, outpatient and/or telephone follow-up was performed. The recurrence rate was observed and VAS > 2 was regarded as recurrence.

**Results:**

At one week after primary treatment, the effective rate was 66.7% in PL group, significantly higher than that in PA group (15.2%, *P* < 0.05). At one week after secondary treatment, the effective rate was 100% and 37.5% in PL and PA groups, respectively, with significant difference between the groups (*P* < 0.05). The effective rate increased to 90.6% in PA group at one week after tertiary treatment. At one week after the end of treatment cycles, the scores of VAS and MPQ were significantly lower in C1, PL, and PA groups than in C2 group (*P* < 0.05), while PPST and PPTT were significantly higher than in C2 group (*P* < 0.05). There was no significant difference between C1 group and PL group (*P* > 0.05). At follow-up at 3 months after treatment, the recurrence rate was low in each group, with no significant difference between the groups (*P* > 0.05).

**Conclusion:**

About 57% of PHN patients with mild to moderate pain are complicated with MPS, and ultrasound-guided inactivation of MTrPs with dry and wet needling can effectively treat PHN patients complicated with LMPS. However, patients with PHN complicated with AMPS need to be treated with ultrasound-guided MTrPs inactivation combined with muscle fascia stripping by liquid knife as soon as possible.

## 1. Introduction

Patients with postherpetic neuralgia (PHN) suffer from persistent and severe breakthrough pain, which may arise from nerve changes due to virus infection or immune response [1, 2]. Some studies have reported that compared with other types of pain, PHN is a neuropathic pain, characterized by severe pain and severe harm to the patient's quality of life [3, 4]. Anticonvulsants, antidepressants, topical therapies, including lidocaine and capsaicin, and opioids are the most widely used therapies for the treatment of PHN [1, 5], but the effect is not very satisfactory. For many years, our routine method for treating PHN in the pain department has been oral medicine and paravertebral nerve block at corresponding spinal cord segments with herpes distribution [1], with good therapeutic effect. However, in the process of diagnosis and treatment, we have found that the nature of pain in some PHN patients overlaps and differs with the classical PHN pain, and our routine treatment shows no ideal effect in these patients. The nature of pain in these PHN patients is characterized not only by neuralgia, such as skin trigger pain, but also by obvious tenderness points, and the pain is not limited to the initial distribution area of herpes, but to a wider range, or even radiating to a distant site. These patients were diagnosed with PHN complicated with myofascial pain syndrome (MPS). MPS is a commonly seen muscle pain disease. It is a clinically common pain syndrome characterized by local muscle pain, accompanied by muscle tension band and involving pain, and may induce local muscle convulsions, and pressing MTrPs can lead to autonomic nerve phenomenon [6, 7]. The diagnostic basis of MPS mainly includes the following [6]. (1) There are hard and striped tension bands in skeletal muscle. (2) There are highly sensitive points (MTrPs) in tension bands, showing blunt pain or sharp pain. (3) There is spontaneous involved pain or involved pain can be induced. (4) Pressing MTrPs leads to recurrence of symptoms, and plucking muscle tension band causes local convulsive response. There are few reports of neuralgia complicated with MPS [8, 9] and they are, if any, only case reports with a small sample size. In 1998, Chen et al. [10] reported that two patients with intercostal nerve PHN had complete pain relief after three times of injection of lidocaine into the intercostal muscle after MPS, with MTrPs found in the intercostal muscle. In 2006, Weiner and Schmader [9] reported another 5 patients with PHN complicated with MPS who were treated with physiotherapy, MTrPs injection, dry needling, and/or transcutaneous neuroelectrical stimulation, and satisfactory results were obtained. In this experiment, a series of treatment measures, such as nerve block, oral medicine, inactivation of MTrPs with dry and wet needling with ultrasound-guided accurate localization, and muscle fascia stripping by liquid knife, were used to treat MTrPs step by step to systematically study the effect in treating PHN complicated with MPS of different sites (limb myofascial pain syndrome, LMPS or abdominal myofascial pain syndrome, AMPS), in order to provide an efficient, feasible, safe, and instructive treatment guide for treating this refractory PHN complicated with MPS.

## 2. Data and Methods

### 2.1. Inclusion Criteria

From January 2015 to July 2018, patients with non-head-and-neck PHN were treated in the Pain Department, The First Affiliated Hospital of Soochow University. Routine treatment was given on the day of initial diagnosis, paravertebral nerve block at corresponding spinal cord segments at the lesion site (once a week), and oral drugs (pregabalin (75∼150 mg, q8h) or gabapentin (300∼600 mg, q8h), amitriptyline (12.5∼25 mg, qn)). After two weeks of routine treatment, those with 2 < VAS < 6 were included in the trial. A total of 256 PHN patients met the preliminary screening criteria, including 110 patients without MPS, 48 PHN patients with MPS, 65 PHN patients with LMPS, and 33 AMPS patients with MPS. Therefore, 33 patients were randomly selected from 110 patients with PHN without MPS, 33 from 48 patients with PHN and MPS, and 33 from 65 patients with PHN and LMPS. A total of 132 patients were enrolled in the experiment. The patients were assigned into 4 groups, control group 1 (C1, *n* = 33) including patients with PHN and without MPS, control group 2 (C2, *n* = 33) including those with PHN and MPS and observation group 1 (PL, *n* = 33) including those with PHN and LMPS (herpes distribution, C5∼T6 and L1∼S1) and observation group 2 (PA, *n* = 33) including those with PHN and AMPS (herpes distribution, T7∼T12). Male or female patients aged 40∼85 years who were clearheaded, had no communication disorder, and were able to take oral medicine were included. Written informed consent for pain treatment was obtained before treatment, and this study was approved by the Ethics Committee of our hospital.

### 2.2. Exclusion Criteria

Patients meeting any of the following criteria were excluded: head-and-neck PHN, obviously abnormal coagulation function, 2 > VAS > 6 after routine treatment for two weeks, mental disorder and disturbance of consciousness, digestive tract obstruction or inability to take oral medicine, serious cardio- or cerebrovascular disease, or heart, liver, kidney and lung failure, allergic to ropivacaine, pregabalin, amitriptyline, gabapentin or any of their excipients, treatments other than those in this study, and fainting during needling or could not tolerate other treatments in this study.

Necessary imaging and other examinations were performed in all patients before enrollment in the study, including routine blood and urine tests, amylase assay, X-ray, CT, and B ultrasound, to exclude pain caused by internal, surgical, and gynecological diseases.

### 2.3. Treatment Methods

#### 2.3.1. Oral Drugs

Pregabalin (Pfizer Pharmaceutical Co., Ltd., 75 mg/tablet), gabapentin capsule (Jiangsu Enhua Pharmaceutical Group Co., Ltd., 0.3 g/tablet) and amitriptyline tablets (Changzhou Siyao Pharmaceuticals Co., Ltd., 25 mg/tablet).

#### 2.3.2. Drugs for Injection

Ropivacaine hydrochloride (AstraZeneca AB, 75 mg/vial), Diprospan (Schering-Plough Labo N.V., 1 ml/vial), and 0.9% sodium chloride injection.

*(1) Formula for Paravertebral Nerve Block and Wet Needle (Formula 1)*. Diprospan 1/2 (containing betamethasone dipropionate 2.5 mg and betazone sodium phosphate 1 mg), 0.75% ropivacaine hydrochloride 4 ml, diluted to 20 ml with 0.9% sodium chloride injection. At a dose of 8 ml for each paraspinal block, paravertebral block of a maximum of two segments was performed *t* at one time. The method of paravertebral nerve block is omitted.

*(2) Formula for Liquid Knife (Formula 2)*. 0.75% ropivacaine hydrochloride 4 mL diluted to 20 ml with 0.9% sodium chloride injection.

The needle used in the study was dental no. 5 long needle (Shanghai Liangzhi Medical Co., Ltd.), and the ultrasonic diagnostic instrument was manufactured by Sonosite Company of the United States, with a probe frequency of 6 × 13 MHz.

#### 2.3.3. Ultrasound-Guided Inactivation of MTrPs with Dry and Wet Needles

For intervention to increase the accuracy of inactivation of MTrPs with dry and wet needle, ultrasound imaging was used to avoid unintentional damage to these important structures, with no irradiation delivered to the patients. The body was examined in advance and marked on the skin with definite tenderness points. The mark points were located in the center of the ultrasonic probe and guided in real time by in-plane technology. The skin was sterilized by Hexial 2% solution (ethanol + chlorhexidine gluconate 2%), and a sterile surgical towel was placed on the patient. A linear array transducer probe was used to scan the marked area in the sagittal and axial planes. Initially, the dental long needle was used to identify the muscle and its fascia. MTrPs showed oval, locally heterogeneous hypoechoic or significant hyperechoic region under two-dimensional ultrasound. The MTrPs were punctured repeatedly with dental needle No. 5 under the guidance of ultrasound to induce obvious aching and distending pain, with no focus on the induction of local twitch responses (LTRs), especially in patients with AMPS, but if LTRs could be induced, the points were punctured as many times as possible until LTRs could not be induced.

The standard of MTrPs inactivation with dry needling, aching, and distending pain induced by needling of MTrPs disappeared or were significantly alleviated, and LTRs disappeared.

**Figure 1:**
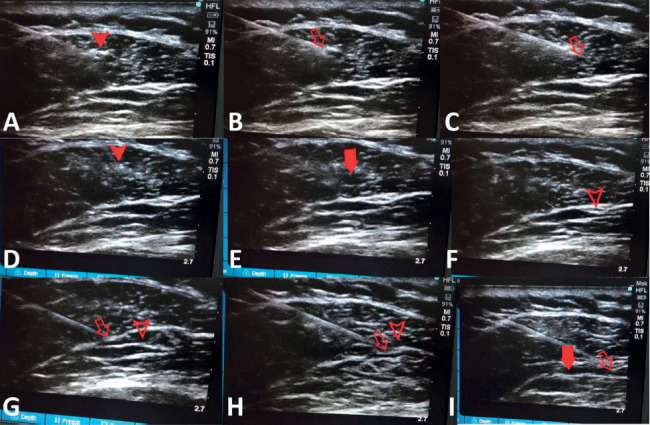
Ultrasound-guided inactivation of trigger points and muscle fascia stripping by liquid knife. The body was examined in advance and marked on the skin with definite tenderness points. The marking points were located in the center of the ultrasonic probe and guided in real time by in-plane technology. (a) Solid triangle refers to the trigger point of the muscle. (b, c) Hollow arrow indicates the tip of the needle, and the trigger point was punctured repeatedly until the patient's aching and distending pain was significantly alleviated and the induced muscle convulsion disappeared. (d) After inactivation of the trigger point, the local echo brightness decreased. (e) Wet needle: liquid of 1 ml was injected into the trigger point, and the solid arrow represents the injected liquid. (f) Hollow triangle refers to muscle fascia. (g) Muscle fascia stretched out by itself when touched by the needle tip. (h) Needle tip pierced into the muscle fascia. (i) Muscle fascia was stripped by the liquid knife, and the solid arrow represents area in the muscle fascia with liquid injected.

Wet needle, after inactivating MTrPs with dry needling, Formula 1 was drawn with 2 ml syringe and injected into and around MTrPs, 1 ml per MTrP, and up to 5 MTrPs were injected at a time (see Figure 1).

#### 2.3.4. Ultrasonic-Guided Muscle Fascia Stripping with Liquid Knife

The preparatory steps used in the treatment of muscular fasciae stripping by liquid knife were identical to those described above of the dry and wet needle inactivation trigger point. The body was examined in advance and marked on the skin with definite tenderness points. The mark points were located in the center of the ultrasonic probe and guided in real time by in-plane technology. The needle tip passed through the muscle fascia with obvious tenderness, and the muscle fascia space which showed significant aching and distending pain or convulsions induced by needling was taken as the target site. First, the muscle fascia was punctured repeatedly until pain disappeared or was significantly alleviated and local convulsions resolved, and then Formula 2 was injected. The muscle fascia was stripped with the liquid. 3∼5 ml was injected into each site, and the muscle fascia was stripped as much as possible, with injection of up to three sites at a time (see Figure 1).

All treatments and interfascial injection were administered by the same physician, but assessments and follow-up were conducted by other investigators who were not aware of group assignments.

#### 2.3.5. Specific Treatment Process and Effect Evaluation of the Whole Experiment

All groups received zero-grade treatment, routine oral drugs and weekly paraspinal nerve block. PL and PA groups also received weekly step-by-step treatment. The first level was ultrasound-guided inactivation of MTrPs. Secondary treatment was ultrasound-guided inactivation of MTrPs with dry and wet needling. Tertiary treatment included ultrasound-guided dry and wet needling combined with muscle fascia stripping by liquid knife. One week after the first-level treatment, the patient's VAS score was >2 points, and the second-level treatment was entered. If the VAS score was <2 points, the current level of treatment was maintained, and so on. The treatment period was four weeks.

*(1) Evaluation Tool*. Pain assessment was performed by specialized nurses at one week after each treatment, including VAS score, MPQ score, PPST and PPTT. The VAS score was critical, with VAS <2 indicating effective treatment and VAS >2 indicating ineffective treatment, and the effective rate was calculated as the number of patients responding to treatment/total number of patients treated. At 3 months after the end of treatment cycles, outpatient and/or telephone follow-up was performed to observe the recurrence rate. VAS >2 was regarded as recurrence in patients responding to treatment previously, and the recurrence rate was calculated as the number of patients experiencing recurrence/the number of patients responding to treatment at one week after the end of treatment cycles.

*(2) VAS*. The VAS was a 10 cm horizontal line labeled no pain at one end and worst imaginable pain at the other end. The patients were asked to mark on this line where the intensity of the pain existed. The distance from no pain to the patients' mark numerically quantified the pain. The VAS was a simple and efficient method that correlated well with other reliable methods.

*(3) McGill Pain Questionnaire (MPQ)*. The MPQ had three parts; the first assessed pain quality and yielded a sensory score (sum of 11 adjectives, throbbing, shooting, stabbing, sharp, cramping, gnawing, hot burning, aching, heavy, tender, and splitting, each rated on an intensity scale with 0 = none, 1 = mild, 2 = moderate, and 3 = severe), an affective score (sum of four adjectives, tiring-exhausting, sickening, fearful, and punishing-cruel related on the same intensity scale), and a total score (sum of the sensory and effective scores). The second part of the short form (SF)-MPQ consisted of a 100 mm VAS of pain intensity that patients used to rate their pain during the preceding week. The third part of the SF-MPQ was a measure of present pain intensity (PPI) using a 6-point scale (0 = none, 1 = mild, 2 = discomfort, 3 = distressing, 4 = horrible, and 5 = excruciating).

*(4) Tenderness Threshold Measurement*. After palpation, the test end of the pressure pain tester (FDK20, Wagner Instruments, USA) was used to gradually contact the skin and was required to be perpendicular to the skin. PPST and PPTT were measured. The peak mode was adopted in the test, and the unit of the scale was kg/cm^2^ with a measurement range of 1∼10 kg/cm^2^. PPST was the level of pressure that subjects felt with the change from pressure to pain. PPTT was the maximum pressure that subjects could tolerate. Usually a maximum value was set, and when pressure reached the value, test was stopped even if the subject still tolerated the pressure, and the maximum value was regarded as PPTT. Measurement was performed for a total of 5 times at an interval of 10∼15 s, and the mean was taken as pain threshold.

*(5) Adverse Reactions*. The adverse reactions related to treatment were truthfully recorded, including nausea, vomiting, lethargy, subcutaneous bleeding, allergic reactions, panic, and dizziness.

#### 2.3.6. Statistical Analysis

All data were analyzed using SPSS 23.0 (IBM Corporation, Armonk, NY, USA). At different time points, there are individual patients falling out, and the data of the patients falling out are not counted. The data were presented as the mean ± SD or *n* (%). The independent-samples *t*-test was used for statistical analysis of the continuous data, and the Chi-squared test was used for statistical analysis of the categorical data. The repeated-measures analysis of variance (ANOVA) test was used to compare data between the two groups before and after treatment. *P* value <0.05 was considered to represent a statistically significant difference.

## 3. Results

### 3.1. Comparison of General Information on Patients between the Four Groups

There were no significant differences in age, gender, weight, duration of PHN, cardiac disease, pulmonary disease, liver and renal diseases, metabolism, and nutrition disorders between the four groups (*P* > 0.05). Results were presented in Table 1.

### 3.2. Comparison of Effective Rate between PL and PA Groups at One Week after Treatment

At one week after primary treatment, the effective rate was 66.7% in PL group, significantly higher than that in PA group (15.2%, *P* < 0.05). At one week after secondary treatment, the effective rate was 100% and 37.5% in PL and PA groups, respectively, with significant difference between the groups (*P* < 0.05), and the effective rate in PA group increased to 90.6% at one week after tertiary treatment. See Table 2.

### 3.3. Comparison of VAS, MPQ, PPST, and PPTT in Each Group at One Week after the End of Treatment Cycles

At one week after the end of treatment cycles, the scores of VAS and MPQ in the four groups were significantly lower than those before treatment (*P* < 0.05), while PPST and PPTT were significantly higher than those before treatment (*P* < 0.05). The scores of VAS and MPQ were significantly lower in C1, PL, and PA groups than in C2 group (*P* < 0.05), while PPST and PPTT were significantly higher than those in C2 group (*P* < 0.05), but there was no significant difference between C1 and PL groups (*P* > 0.05). The scores of VAS and MPQ were slightly higher in PA group than those in C1 and PL groups, while PPST and PPTT were slightly lower than those in C1 and PL groups, with no significant difference (*P* > 0.05). See Table 3.

### 3.4. Comparison of Efficacy in Each Group at Follow-Ups at One Week and Three Months after the End of Treatment Cycles

At follow-ups at one week and three months after the end of treatment cycles, the effective rate was significantly higher in C1, PL, and PA groups than in C2 group (*P* < 0.05). Among the former three groups, the effective rate was slightly higher in PL group than in the other two groups, but there was no significant difference (*P* > 0.05). At follow-up at 3 months after the end of treatment cycles, a very small number of patients in each group had recurrence, with the lowest recurrence rate in C1 group (3.8%). The recurrence rate was similar in the other three groups, but there was no significant difference among the four groups (*P* > 0.05). See Table 4.

### 3.5. Adverse Reactions Observed in Four Groups after Treatment

Finally, in this study, adverse reactions were also observed, including nausea, vomiting, dizziness, somnolence, palpitations, chest tightness, bleeding, infection, and allergy in the four groups after treatment. The results showed that the adverse effects experienced in the four groups were not statistically different after treatment (*P* > 0.05), as shown in Table 5.

## 4. Discussion

PHN is the most common and intractable complication of herpes zoster, and it is a serious neuropathy of peripheral nerves and central nervous system caused by herpes zoster virus infection. Clinical manifestations are peripheral neuralgia and sensory abnormalities such as allodynia [3]. There is no unified standard for the definition of PHN time. Most scholars believe that PHN can be diagnosed if the duration of neuralgia exceeds 90 days [11]. About 10% of patients with herpes zoster are complicated with PHN, with an incidence of 50%∼70% in elderly patients over 60 years of age [4]. This refractory neuropathic pain causes extreme psychological and physical pain to the patients, leading to a serious impact on the quality of life. So far, however, there has been no reliable and effective way to eliminate this neuralgia, and many patients in whom PHN treatment is effective are prone to pain recurrence [12]. For many years, in our pain department, the conventional method for the treatment of PHN is that PHN with mild to moderate pain (6 > VAS > 2) is treated with paravertebral nerve block in spinal cords segments corresponding to herpes distribution area combined with oral drugs; patients with severe pain (VAS > 6) or patients in whom the above treatment is ineffective are treated with epidural or intrathecal analgesia pump [13], spinal cord electrical stimulation [14], transcutaneous electrical stimulation, nerve root radiofrequency thermocoagulation or pulse radiofrequency ablation [15], etc. This study focuses on the treatment of PHN patients with mild to moderate pain. In this study, it was reported for the first time that more than 50% of PHN patients with mild to moderate pain had MPS. It was found that routine treatment showed ideal therapeutic effect in PHN patients without MPS, but this was not the case in patients with PHN complicated with MPS. For these patients, combination with treatment of MPS had unexpectedly good results, especially in patients with PHN complicated with LMPS, with an effective rate of almost 100%.

MPS is a common clinical disease caused by MTrPs on muscle fascia. The mechanism of MTrPs formation is not very clear. It is generally believed that [16] MTrPs may be caused by muscle trauma, long-term incorrect posture, or repeated local injury. They can be divided into active trigger points (ATrPs) and latent trigger points (LTrPs). LTrPs have all of the other characteristics of ATrPs except for causing no spontaneous pain. LTrPs may turn into ATrPs due to certain diseases, such as accumulated muscle strain, poor posture, systemic diseases, and neuromuscular and skeletal diseases. At present, hypotheses about the formation mechanism of MTrPs are as follows: the energy metabolism crisis put forward by Simons and Travell in 1980 [6], the theory of abnormal potential of muscle spindle by Barnes in 1996 [17], and the theory of abnormal function of motor endplate by Mense et al. [18]. It is also proposed that MPS is related to peripheral and central sensitization of pain [19, 20]. The pain of PHN is characterized by significant allodynia in herpes distribution area, manifested by severe hyperalgesia caused by temperature changes due to light touch, blowing wind, cold, or warmth. This intractable and intense neuralgia is closely related to peripheral and central sensitization of pain [21]. We speculate that the reasons for such a high proportion of PHN patients with MPS (about 57%) are as follows: first, the passive protective posture of PHN patients due to pain, for a prolonged time, which will lead to cumulative muscle strain and is a hotbed for formation of MTrPs; second, erosion of the nervous system by virus in PHN patients and the resulting central and peripheral sensitization of pain, which activates LTrPs already present in these patients and causes concomitant MPS. In other words, we believe that PHN can lead to MPS, with a quite high incidence. The latest study by Do et al. [22] also confirmed that MPS was common in patients with migraine and tension headache and was a reaction secondary to and not a cause of this neuralgia. The pain of patients with PHN complicated with MPS is characterized by both allodynia and definite tenderness points, and the pain is not limited to the distribution area of initial herpes, but to a wider range, or even radiating to a distance site. This may be the basic reason why routine treatment shows a poor effect for neuralgia in patients with this type of PHN.

In the past 20 years, diagnosis of MTrPs has been subject to continuous revision according to clinical practice and research, but up to now, there is still no objective and reliable standard. Therefore, the objective and reliable detection and efficacy evaluation of MTrPs by modern physical technology is of great clinical significance for the diagnosis and treatment of MPS. At present, the main methods for detecting MTrPs are as follows: thumb palpation (subjects are asked to tell if there is tension and pain during manual pressing. MTrPs can be diagnosed by positive reaction), tenderness threshold detected by pain detector, ultrasound imaging, electromyography (EMG), magnetic resonance elastography (MRE), thermal texture maps (TTM), etc. [23, 24]. Ultrasound is the most widely used in the diagnosis and treatment of MTrPs. It is suggested in study that MTrPs are oval hypoechoic regions [22] by two-dimensional ultrasonography, but it is also believed that MTrPs are hyperechoic regions [25]. There is also study demonstrating that ultrasound is of great significance to the detection of LTRs in patients with MTrPs, especially for deep tissues [26]. In this study, the following three steps were used to diagnose MTrPs. The first step was to examine the body in advance, to carefully palpate in the area where the pain might be present, and to initially identify MTrPs, manifested as the patient's obvious tenderness, involved pain and local convulsions, and mark the corresponding skin. In the second step, the marking point was placed in the center of the ultrasonic probe, and the heterogeneous area (hypoechoic or hyperechoic area) in the muscle or fascia was determined by two-dimensional ultrasound scanning. In the third step, using in-plane technique, under ultrasound real-time guidance, dental needle No.5 was used to puncture the MTrPs area, and MTrPs were confirmed when patients had obvious aching and distending pain and/or LTRs were induced. Then, MTrPs were punctured repeatedly, until the pain disappeared or was significantly alleviated and LTRs disappeared, indicating MTrPs inactivation. It is worth mentioning that a lot of clinical experience suggests that by two-dimensional ultrasound imaging, most of the MTrPs in the muscle are local high-brightness areas, while those in the muscle fascia are mainly hypoechoic areas in PHN patients with MPS. Moreover, stimulation of MTrPs in the muscle can mostly induce LTRs, while stimulation of MTrPs in the muscle fascia shows discomfort including obvious local aching and distending pain. Therefore, in treatment, we do not focus on inducing LTRs in inactivating MTrPs [27]. However, if they can be induced, the MTrPs are stimulated until LTRs cannot be induced.

At the same time, it is interesting to note that when the needle tip stimulated the MTrPs on the muscle fascia, the tangled muscle fascia stretched out instantly on the ultrasound image in some patients (see Figure 1(g)). Treatment effect was better in such patients with significant muscle fascia stretching. The occurrence of this phenomenon may be explained by the formation of local MTrPs, leading to changes inform and structure or wrinkle of the muscle and muscle fascia, resulting in compression of the piercing nerve and blood vessels, causing pain. To a certain extent, pain can affect the movement mode of muscle and fascia, which leads to the formation of MTrPs in muscle fascia, and there is a causal relationship between them. Needling acts exactly on MTrPs, significantly improving deformation or wrinkle of the points, which becomes the clinical basis for the treatment of PHN complicated with MPS [28]. In contrast, we also observed a significant decrease in the echo of the high-brightness area after inactivation of MTrPs in the muscle (see Figure 1(d)).

Furthermore, we explored the effective treatment measures for PHN patients complicated with LMPS or AMPS, respectively. The results showed that the therapeutic effect of dry and wet needling was not as good in PA group as in PL group. The therapeutic effect was satisfactory only after combination with muscle fascia stripping by liquid knife. The difference in therapeutic effect may be due to the fact that in patients complicated with LMPS, stimulation of MTrPs in the muscle can mostly induce LTRs because of well-developed muscles of the limbs, which is enough to unfold the deformed and wrinkled muscle fibers, in turn, which interrupts the vicious circle of pain (PHN)-MPS pain. However, the abdominal muscle is relatively weak and usually covered by muscle fascia. Stimulation of MTrPs in the muscle fascia can generally not induce LTRs, and deformed and wrinkled muscle fascia can only stretch out with the help of physical stripping effect of the liquid knife.

In addition, it is confirmed that local anesthetic injection in MTrPs can regulate the central sensitization of chronic pain [29], but it is also believed that local anesthetic injection in MTrPs can only be used in the diagnosis of MTrPs and cannot achieve enduring relief of pain. Therefore, steroid injection or pulse radiofrequency regulation of MTrPs is recommended [30]. Meanwhile, considering that repeated stimulation of MTrPs with dry needling may lead to prolonged postoperative local pain [31], we recommend that a small dose of the mixture of local anesthetic and hormone be injected locally after inactivation of MTrPs with dry needling to reduce the pain score of patients with PHN complicated with MPS and maintain the analgesic effect, with a low recurrence rate at follow-up after three months.

Limitations of this study are as follows. (1) The duration of follow-up in this study is not long enough, and only the recurrence rate at 3 months after the last treatment was followed up. In view of the intractable and refractory characteristics of PHN, observation and follow-up for a longer time will be performed in our further study. (2) The time point for assessment of treatment effect is one week after each treatment, which makes it difficult to tell whether the assessment results are cumulative effect of the previous treatment or effect of the new treatment method. (3) Substudy is not performed on duration of PHN because of the small sample size. Although the mean duration of PHN in each group is not statistically significant, clinical experience and literature research have told us [11] that the difficulty in treatment of PHN will increase over time. Therefore, it is necessary to investigate the differences in duration and therapeutic effect of PHN.

## 5. Conclusions

In summary, the trigger point related theory holds that tenderness amplification and involved pain caused by MPS are the important causes of chronic pain, and it is confirmed that MPS is a major complication of neuralgia. Therefore, patients with PHN complicated with MPS should be considered carefully in clinic. It will be better to start with the treatment of MTrPs in the presence of MPS. In this study, about 57% of PHN patients with mild to moderate pain are complicated with MPS, and ultrasound-guided inactivation of MTrPs with dry and wet needling can effectively treat patients with PHN complicated with LMPS. However, patients with PHN complicated with AMPS need to be treated with ultrasound-guided inactivation of MTrPs with dry and wet needling combined with muscle fascia stripping by liquid knife as soon as possible.

## Figures and Tables

**Table 1:** Characteristics of four groups at baseline ($\bar{x}\pm s$, *n*).

Characteristic	C1 (*N* = 33)	C2 (*N* = 33)	PL (*N* = 33)	PA (*N* = 33)
Age (years)	61.3 ± 10.3	62.1 ± 9.6	62.4 ± 12.7	60.9 ± 13.4
Gender (male/female)	17/16	15/18	14/19	13/20
Weight (kg)	59.4 ± 18.2	61.4 ± 20.5	58.9 ± 17.6	62.1 ± 19.8
Duration of PHN (months)	7.9 ± 2.5	8.2 ± 3.1	7.8 ± 2.2	8.1 ± 2.2
Cardiac disease	8/33	10/33	9/33	10/33
Pulmonary disease	15/33	16/33	14/33	17/33
Liver and renal diseases	8/33	9/33	7/33	8/33
Metabolism and nutrition disorders	17/33	16/33	16/33	19/33

**Table 2:** Comparison of the efficient of PL and PA groups one week after graded treatment.

Group	Efficient at 1 week after primary treatment (%, *n*)	Efficient at 1 week after secondary treatment (%, *n*)	Efficient at 1 week after tertiary treatment (%, *n*)
PL	66.7 (22/33)	100 (33/33)	100 (32/32)
PA	15.2 (5/33)^a^	37.5 (12/32)^a^	90.6 (29/32)

*Note*. ^a^Significantly different compared to the PL group, *P* < 0.05.

**Table 3:** VAS, MPQ, PPST, and PPTT score of four groups before treatment cycle and 1 week after the end of treatment cycle ($\bar{x}\pm s$, *n*).

Characteristic		C1 (*N* = 31)		C2 (*N* = 32)		PL (*N* = 32)		PA (*N* = 32)	
		T0	T1	T0	T1	T0	T1	T0	T1
VAS score		4.8 ± 1.4	1.6 ± 0.5^a,b^	4.7 ± 1.6	3.1 ± 1.1^a^	5.0 ± 1.9	1.7 ± 0.6^a,b^	4.6 ± 1.3	2.0 ± 0.4^a,b^
MPQ	Sensory score	4.2 ± 1.1	2.1 ± 1.0^a,b^	4.6 ± 1.9	3.5 ± 0.7^a^	4.4 ± 1.2	2.0 ± 0.8^a,b^	4.5 ± 1.5	2.5 ± 0.5^a,b^
	Effective score	3.1 ± 0.4	1.3 ± 0.5^a,b^	3.3 ± 0.7	2.3 ± 0.5^a^	3.2 ± 0.9	1.4 ± 0.3^a,b^	3.2 ± 1.1	1.5 ± 0.9^a,b^
	Total score	6.8 ± 2.4	3.4 ± 1.3^a,b^	6.9 ± 2.2	5.1 ± 1.8^a^	7.1 ± 2.6	3.5 ± 1.4^a,b^	7.2 ± 2.7	4.0 ± 1.7^a,b^
	VAS (mm)	46.8 ± 11.4	18.6 ± 6.5^a,b^	47.5 ± 14.5	39.6 ± 16.8^a^	50.1 ± 13.7	17.1 ± 8.6^a,b^	48.9 ± 12.6	22.1 ± 9.9^a,b^
	PPI	3.5 ± 1.3	1.2 ± 0.7^a,b^	3.7 ± 1.6	2.5 ± 0.8^a^	3.8 ± 1.4	1.2 ± 0.9^a,b^	3.9 ± 1.9	1.6 ± 0.6^a,b^
PPST (kg/cm^2^)		1.6 ± 0.4	4.8 ± 1.8^a,b^	1.5 ± 0.8	3.5 ± 1.6^a^	1.7 ± 0.9	4.7 ± 1.4^a,b^	1.6 ± 0.6	4.1 ± 1.3^a^
PPTT (kg/cm^2^)		2.3 ± 0.8	5.7 ± 2.1^a,b^	2.2 ± 0.5	4.0 ± 1.1^a^	2.4 ± 0.9	5.3 ± 1.9^a,b^	2.2 ± 0.7	5.0 ± 1.9^a,b^

*Note*. T0 = before treatment cycle. T1 = 1 week after the end of treatment cycle. ^a^Significantly different compared to the T0 group, *P* < 0.05. ^b^Significantly different compared to the C2 group, *P* < 0.05.

**Table 4:** Comparison of the efficacy of each group at one week after the end of treatment cycle and after three months of follow-up.

Group (*n*)	Efficient at 1 week after the end of treatment cycle (%, *n*)	Efficient at three months of follow-up (%, *n*)	Recurrence rate at three months of follow-up (%, *n*)
C1	83.9 (26/31)^a^	79.3% (23/29)^a^	3.8% (1/26)
C2	37.5 (12/32)	33.3% (10/30)	16.7% (2/12)
PL	96.9 (31/32)^a^	89.7 (26/31)^a^	12.9% (4/31)
PA	87.5 (28/32)^a^	75.9 (22/29)^a^	10.7% (3/28)

*Note*. ^a^Significantly different compared to the C2 group, *P* < 0.05.

**Table 5:** Adverse reactions observed in four groups after treatment.

Adverse reaction	C1 (*N* = 33)	C2 (*N* = 33)	PL (*N* = 33)	PA (*N* = 33)
Nausea	3/33	2/33	1/33	2/33
Vomiting	0/33	0/33	1/33	1/33
Dizziness	3/33	4/33	4/33	5/33
Somnolence	5/33	6/33	7/33	5/33
Palpitation	2/33	1/33	1/33	1/33
Chest tightness	3/33	2/33	1/33	3/33
Bleeding	1/33	0/33	1/33	0/33
Infection	0/33	0/33	0/33	0/33
Allergy	1/33	0/33	0/33	0/33

## Data Availability

The data were detected in November 2018. The data used to support the findings of this study are available from the corresponding author upon request.

## Abbreviations

PHN: Postherpetic neuralgia
MPS: Myofascial pain syndrome
P: Paravertebral block
O: Oral drugs
D: Dry needle
W: Wet needle
L: Liquid knife.
